# Wound Infections Following Implant removal below the knee: the effect of antibiotic prophylaxis; the WIFI-trial, a multi-centre randomized controlled trial

**DOI:** 10.1186/1471-2482-15-12

**Published:** 2015-02-06

**Authors:** Manouk Backes, Siem A Dingemans, Niels WL Schep, Frank W Bloemers, Bart Van Dijkman, Frank P Garssen, Robert Haverlag, Jochem M Hoogendoorn, Pieter Joosse, Boj Mirck, Victor Postma, Ewan Ritchie, W Herbert Roerdink, Jan Bernard Sintenie, Nicolaj MR Soesman, Nico L Sosef, Bas A Twigt, Ruben N Van Veen, Alexander H Van der Veen, Romuald Van Velde, Dagmar I Vos, Mark R De Vries, Jasper Winkelhagen, J Carel Goslings, Tim Schepers

**Affiliations:** Trauma Unit, Department of Surgery, Academic Medical Center, University of Amsterdam, P.O. Box 22660, 1100 DD Amsterdam, The Netherlands; Traumasurgery, Department of Surgery, VU University Medical Center, P.O. Box 7057, 1007 MB Amsterdam, The Netherlands; Department of Surgery, Flevo Hospital, P.O. Box 3005, 1300 EG Almere, The Netherlands; Department of Surgery, Hospital Amstelland, P.O. Box 328, 1180 AH Amsterdam, The Netherlands; Department of Surgery, Onze Lieve Vrouwe Hospital, P.O. Box 95500, 1090 HM Amsterdam, The Netherlands; Department of Surgery, MC Haaglanden, P.O. Box 432, 2501 CK The Hague, The Netherlands; Department of Surgery, Medical Center Alkmaar, P.O. Box 501, 1800 AM Alkmaar, The Netherlands; Department of Surgery, Red Cross Hospital, P.O. Box 1074, 1940EB Beverwijk, The Netherlands; Department of Surgery, MC Zuiderzee, P.O. Box 5000, 8200 GA Lelystad, The Netherlands; Department of Surgery, Rijnland Hospital, P.O. Box 4240, 2350 CC Leiderdorp, The Netherlands; Department of Surgery, Deventer Hospital, P.O. Box 5001, 7400 GC Deventer, The Netherlands; Department of Surgery, Elkerliek Hospital, P.O. Box 98, 5700 AB Helmond, The Netherlands; Department of Surgery, Vlietland Hospital, P.O. Box 215, 3100 AE Schiedam, The Netherlands; Department of Surgery, Spaarne Hospital, P.O. Box 770, 2130 AT Hoofddorp, The Netherlands; Department of Surgery, BovenIJ Hospital, PO Box 37610, 1030 BD Amsterdam, The Netherlands; Department of Surgery, Sint Lucas Andreas Hospital, PO Box 9243, 1006 AE Amsterdam, The Netherlands; Department of Surgery, Catharina Hospital, P.O. Box 1350, 5602 ZA Eindhoven, The Netherlands; Department of Surgery, Tergooi Hospitals, P.O. Box 10016, 1201 DA Hilversum, The Netherlands; Department of Surgery, Amphia Hospital, P.O. Box 90157, 4800 RL Breda, The Netherlands; Department of Surgery, Reinier de Graaf Hospital, P.O. Box 5011, 2600 GA Delft, The Netherlands; Department of Surgery, Westfries Hospital, P.O. Box 600, 1620 AR Hoorn, The Netherlands

**Keywords:** Antibiotic prophylaxis, Postoperative wound infection, Implant removal, Fracture surgery, Functional outcome

## Abstract

**Background:**

In the Netherlands about 18,000 procedures with implant removal are performed annually following open or closed reduction and fixation of fractures, of which 30-80% concern the foot, ankle and lower leg region. For clean surgical procedures, the rate of postoperative wound infections (POWI) should be less than ~2%. However, rates of 10-12% following implant removal have been reported, specifically after foot, ankle and lower leg fractures. Currently, surgeons individually decide if antibiotics prophylaxis is given, since no guideline exists. This leads to undesirable practice variation. The aim of the study is to assess the (cost-)effectiveness of a single intravenous gift of Cefazolin prior to implant removal following surgical fixation of foot, ankle and/or lower leg fractures.

**Methods:**

This is a double-blind randomized controlled trial in patients scheduled for implant removal following a foot, ankle or lower leg fracture. Primary outcome is a POWI within 30 days after implant removal. Secondary outcomes are quality of life, functional outcome and costs at 30 days and 6 months after implant removal. With 2 x 250 patients a decrease in POWI rate from 10% to 3.3% (expected rate in clean-contaminated elective orthopaedic trauma procedures) can be detected (Power = 80%, 2-sided alpha = 5%, including 15% lost to follow up).

**Discussion:**

If administration of prophylactic antibiotics prior to implant removal reduces the infectious complication rate, this will offer a strong argument to adopt this as standard practice of care. This will consequently lead to less physical and social disabilities and health care use. A preliminary, conservative estimation suggests yearly cost savings in the Netherlands of € 3.5 million per year.

**Trial registration:**

This study is registered at Clinicaltrials.gov (NCT02225821) and the Netherlands Trial Register (NTR4393) and was granted permission by the Medical Ethical Review Committee of the Academic Medical Centre on October 7 2014.

## Background

Open or closed reduction followed by internal fixation is a frequently performed operation for lower extremity fractures. Indications for implant removal in adult patients include symptomatic hardware (i.e. pain, thin overlying skin and restricted motion), implant failure (breakage, loosening), or a persistent infectious complication of the index procedure (infection or fistula). Following successful surgical procedures for extremity fractures, implant removal is not a routinely indicated procedure. However, removal of implants causing symptoms can result in pain relief and a high rate of patient satisfaction [1, 2].

In the Netherlands about 18,000 implant removals are performed annually, of which 30-80% in the foot, ankle and lower leg region [3]. Literature on implant removal is sparse, but studies show most of the implants removed are following lower extremity injuries, especially below the knee (Table 1).

**Table 1:** **Studies on implant removal and the portion of implant removal from the foot**-**ankle and lower leg region**

Study (year)	N of cases	N of IR FAL (%)
Raahave (1967) [4]	269	109 (41)
Richards (1992) [5]	88	25 (28)
Sanderson (1992) [6]	188	92 (49)
Minkowitz (2007) [7]	60	42 (70)
Vos (2012) [2]	284	89 (31)
Backes (2015) [8]	512	404 (79)

*N*; Number, *IR*; implant removal, *FAL*; foot- ankle or lower leg.

In addition, there is only a small amount of literature available on the risk of postoperative wound infection (POWI) following implant removal (Table 2). For ‘clean’ procedures the rate of POWI should be less than ~2% [11]. However, POWI rates of about 10-12%, specifically after foot, ankle and/or lower leg fractures, have been observed both by us and others in studies in which patients with implant removal due to an active wound infection were excluded [2, 8]. In syndesmotic screw removal 9.2% of POWI were observed and in calcaneal implant removal following fracture surgery without postoperative complications in dislocated closed calcaneal fractures 19% of POWI were observed [9, 10]. Preoperative prophylactic antibiotics might be beneficial to reduce the incidence of infectious complications following implant removal.

**Table 2:** **Implant removal and incidence postoperative wound infections**

Study (year)	N of cases	N of IR in FAL	N of POWI in FAL (%)
Raahave (1967) [4]	269	109	4 (3.7)
Richards (1992) [5]	88	25	0 (0)
Sanderson (1992) [6]	188	92	12 (13)
Minkowitz (2007) [7]	60	42	0 (0)
Schepers (2011) [9]	76	76	7 (9.2)
Backes (2013) [10]	228	69	6 (9)
Vos (2012) [2]	284	89	9 (11)
Backes (2015) [ [8]	512	403	49 (12.2)

*N*; Number, *IR*; implant removal *NA*; not available, *POWI*; postoperative wound infection, *FAL*; foot- ankle and lower leg.

To date, only evidence exists on the effectiveness of prophylactic antibiotics in internal fixation with implants, but not in implant removal to prevent POWI [12]. In the Netherlands antibiotic prophylaxis is not routinely administered prior to implant removal as it is considered a clean procedure. Surgeons decide upon themselves if antibiotics are administered prior to implant removal, which is based on expert opinion as no evidence based guideline exists. This results in a undesirable practice variation.

Our aim is to study the (cost-)effectiveness of a single intravenous gift of Cefazolin prior to implant removal following surgical fixation of foot, ankle and/or lower leg fractures. The primary outcome is the incidence of POWI and secondary outcomes are health-related quality of life, functional outcome, health care utilization including transmural care, and costs from a health care and societal perspective.

## Methods

This double blind randomised controlled trial will randomise between pre-operative administration of a single gift of Cefazolin or sodium chloride 0.9% in patients scheduled for elective implant removal below the knee. Twenty one centers will participate, including two Level 1 trauma centers.

### Participants

The eligible study population will consist of all consecutive adult patients who are planned for elective implant removal following fracture treatment of the foot, ankle and/or lower leg.

### Inclusion criteria

- Patients ≥18 years and ≤75 years of all ethnic backgrounds
- Scheduled implant removal following foot, ankle and/or lower leg surgery

### Exclusion criteria

- Removal and adding osteosynthesis material during the same procedure
- Active wound infection or (plate) fistula
- Antibiotic treatment at the time of implant removal for a concomitant disease or infection
- A medical history of an allergic reaction to a cephalosporin, penicillin, or any other β-lactam antibiotic
- Known kidney disease (or known eGFR <60 ml/min/1.73 m^2^)
- Pregnancy and lactation
- Immunosuppressant use in organ transplantation or rheumatoid joint disease

### Interventions

After obtaining informed consent in the outpatient clinic, patients are contacted for a pre-operative assessment of functional status and health-related quality of life by way of self-administered questionnaires before surgery.

**Figure 1:**
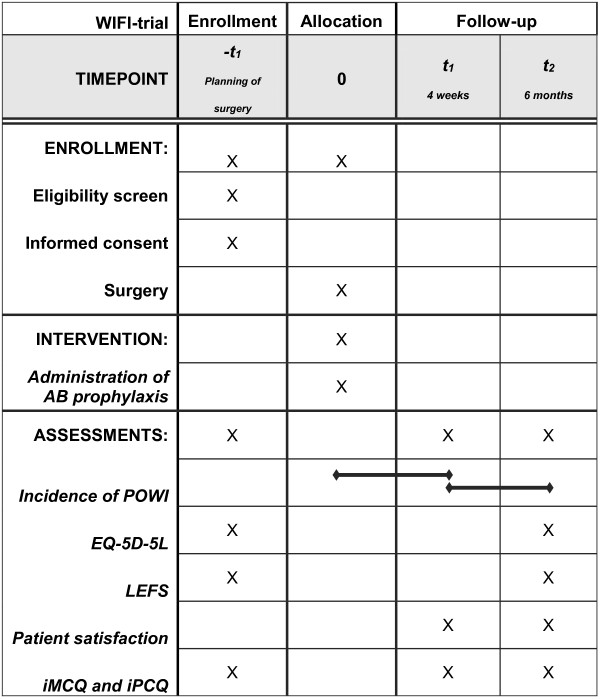
**Schedule of the study procedures.** AB; antibiotic, POWI; postoperative wound infection, EQ-5D; EuroQuality of Life-5D, LEFS; Lower extremity functional Scale, iMCQ; iMTA Medical Consumption Questionnaire, iPCQ; MTA Productivity Cost Questionnaire.

At the day of surgery, patients will be randomly assigned web-based in a 1:1 allocation ratio to one of the following study arms:

1. antibiotic prophylaxis: a single intravenous (iv) gift of 1000 mg Cefazolin in 10 cc of NaCl 0.9% (intervention group) or
2. no antibiotic prophylaxis: a single iv gift of 10 cc NaCl 0.9%.After implant removal, patients are routinely assessed within four weeks postoperatively at the outpatient clinic (Figure 1). They are instructed to visit the outpatient clinic sooner in case of any signs of POWI, including warmth, redness, pain, swelling, drainage or a fever above 38.5 degrees Celsius. In case of a POWI, appropriate treatment is started according to protocol. In addition to the one time visit, the patient is asked to return a surgical wound healing post-discharge questionnaire by mail filled in at thirty days postoperatively. At six months after implant removal, patients are contacted by telephone or mail to fill out web-based questionnaires to assess functional outcome, QOL measurement, patient satisfaction, health care resources utilization, costs evaluation and questions on late infections (Figure 1).

### Randomization

Randomization will be stratified per center and will be blocked within strata. Randomization sequence is generated by a dedicated computer randomization software program and will be performed preoperatively by a theatre assistant and/or the anaesthesiologist using a dedicated, password protected, SSL–encrypted website, ensuring allocation concealment during the Time Out Procedure. Given the randomization result, the anaesthesiologist will prepare either a syringe with 1000 mg Cefazolin or with NaCl 0.9% in the operating theatre or pre-operative holding area, which is administered thirty minutes prior to surgery through a peripheral iv catheter. The iv-catheter is used routinely for either sedatives, muscle relaxants and/or pain medication.

### Blinding

Importantly, the anaesthesiologist prepares the study medication in the absence of the surgeon and administers the study medication or NaCl 0.9%. Neither the patient nor the surgeon will know if the patient receives prophylactic antibiotics. During the visit to the outpatient clinic the patient is seen by a physician other than the surgeon who performed the surgery. The attending physician will document signs of POWI and will determine its presence or any special findings on physical examination. In addition, a photograph of the wound(s) will be taken by the attending physician and kept in the medical charts. This will enable an independent outcome assessment committee to judge the clinical aspect of the surgical wound, blinded for the study intervention. If the local investigator or attending physician decides unblinding is essential, (s)he will make every effort to contact the coordinating investigator before unblinding to discuss options. Otherwise, the randomization code will be unblinded after analysis of the study results.

### Primary Outcome

The primary outcome variable is a POWI within 30 days after implant removal as defined by the criteria applied by the CDC [11].

### Secondary Outcomes

The study will focus on the following secondary outcomes (Figure 1):

- Health-related quality of life as measured by the EQ-5D questionnaire. The EQ-5D-5 L is a descriptive system of health-related quality of life states consisting of five dimensions (mobility, self-care, usual activities, pain/discomfort and anxiety/depression [13].
- Functional outcome as assessed with the Lower Extremity Functional Scale (LEFS). The LEFS is a questionnaire containing 20 questions about a person’s ability to perform everyday tasks and can be used to monitor the patient over time and to evaluate the effectiveness of an intervention [14, 15].
- Patient satisfaction as measured by a ten-point Visual Analog Scale.
- Health care resources utilization (including amongst others, number of visits to the general practitioner and use of home care organizations) as measured by way of a combination of the Dutch iMTA Medical Consumption Questionnaire (iMCQ) and iMTA Productivity Cost Questionnaire (iPCQ).
- Costs (economic evaluation including budget impact analysis): the economic evaluation of antibiotic prophylaxis in patients scheduled for implant removal following a foot, ankle or lower leg fracture against no prophylaxis as its best alternative will be performed as a cost-effectiveness (CEA) as well as a cost-utility (CUA) analysis. The primary economic outcome in the CEA will be the costs per patient without a POWI, which closely relates to the clinical outcome measure. The CUA outcome is the costs per quality adjusted life year (QALY), which is a suitable outcome measure for priority setting during health care policy making across interventions, patient populations, and health care settings.

### Sample size

Since information from prospective studies is limited, there is uncertainty about the POWI rate in current medical practice. In recent Dutch prospective studies the incidence of POWI below the knee is 11%, 12.2%, 9.2% and 19% [2, 8–10]. To be on the safe side, a POWI rate of 10% is assumed for the control group. According to the expected rate in clean-contaminated elective orthopedic procedures, a POWI rate of 3.3% for the antibiotic prophylaxis group is assumed [11]. At least 216 patients per study arm are necessary to detect this difference with a power of 80% and a two-sided alpha of 5%. An estimation of the POWI rate in the control group is planned midway, when 216 patients have been included and reached the primary outcome at 30 days post-surgery. Since only an estimation of the POWI rate of the control group is performed and no treatment effect is tested, the overall Type I error rate is maintained. This estimation will be performed by an independent statistician. To allow for an anticipated drop out of 10-15%, we will include a total of 250 patients per arm.

Based on our recent retrospective cohort studies in both an academic and non-academic hospital an annual number of 33–66 patients are expected to be included in our study for implant removal following lower leg injuries for each participating clinic [8]. With a number of 21 participating centers and an inclusion period of 1.5 years the number of study participants needed, is therefore highly feasible.

### Statistical analysis

All analyses will be performed according to the intention-to-treat principle. In addition, protocol analyses will be done to check for robustness of results. A two-sided P-value < 0.05 will be considered statistically significant. In all analyses statistical uncertainties will be quantified using corresponding 95% two-sided confidence intervals. Descriptive analysis will be performed to compare baseline characteristics between patients with and without an infection. Univariate analysis will be performed for primary and secondary outcomes, followed by a multivariate logistic regression analysis to eliminate confounders. All analyses will be done using the Statistical Package for the Social Sciences (SPSS) version 19.0. (SPSS, Chicago, Illinois, USA).

### Regulation statement

The study will be conducted according to the principles of the Declaration of Helsinki (version 10, 64th WMA General Assembly, Forteleza, Brazil, October 2013) and in accordance with the Medical Research Involving Human Subjects Act (WMO) and the Good Clinical Practice Guidelines (ICH-GCP).

### Recruitment and consent

The patient will be informed about the WIFI-trial when he or she visits the outpatient clinic and implant removal is discussed. Documents are handed to the patient and the patient is asked to read the patient information letter. In order to be able to prepare for the elective (day care) surgery the patient is asked to participate in the trial during this visit to the outpatient clinic and will be asked to sign the informed consent form. Surgeons are asked by the coordinating investigator to check whether patients are included in the pre-operative assessment a day prior to surgery.

### Benefits and risks assessment, group relatedness

Patient risks in this study are minimal and acceptable, as Cefazolin is currently used as prophylaxis in open reduction and internal fixation of fractures. Patients in both study groups will not be exposed to risks other than in current practice, since there is practice variation in the use of prophylactic antibiotics. As mentioned, currently surgeons decide upon themselves if antibiotics are administered preoperatively. We assume that the routine use of prophylactic antibiotics prior to implant removal following surgical fixation of foot, ankle and/or lower leg fractures will reduce the rate of POWI significantly (by two-thirds, from 10% to 3.3%). If our hypothesis is supported by the results of the proposed RCT, this will offer a strong argument to incorporate prophylactic use of a Cefazolin as strategy of choice in (inter)national guidelines for implant removal following fixation of ankle, foot and lower leg fractures. This could lead to less morbidity and social adverse effects in patients like pain, physical discomfort, multiple outpatient clinic visits/less healthcare consumption, work absenteeism and decreased self-confidence.

### Indemnities

The institutional review board at the AMC has waived liability insurance, because no additional risk can be attributed to participation in this study.

### Publication plan

The principal investigator, the study designer and the study coordinator will be named author. There will be a limit of ten authors. All others will obtain group authorship in the study group. All authors including group members are allowed to present the results.

## Discussion

This RCT on wound infections following implant removal is performed in twenty-one different hospitals by a larger number of surgeons, which causes heterogeneity in patients and surgeons. However, we believe this also reflects normal practise in which antibiotic profylaxis could be beneficial. If our assumption that prophylactic antibiotics prior to implant removal reduces the infectious complication rate is confirmed by this RCT, this will offer a strong argument to adopt a single gift of antibiotic prophylaxis as standard practice of care. This will reduce the incidence of POWI and consequently will lead to less physical and social disabilities and health care use. In addition, it will decrease the rate of use of empiric broad-spectrum antibiotics (and antibiotic resistance) prescribed upon suspicion or diagnosis of a POWI. A preliminary, conservative estimation suggests yearly cost savings in the Netherlands of € 3.5 million per year.

## Abbreviations

AB: Antibiotic
CEA: Cost-effectiveness analysis
CUA: Cost-utility analysis
EQ-5D: EuroQuality of Life-5D
FAL: Foot- ankle and lower leg
iMCQ: iMTA Medical Consumption Questionnaire
iPCQ: MTA Productivity Cost Questionnaire
IR: Implant removal
Iv: Intravenous
LEFS: Lower extremity functional Scale
N: Number
NA: Not available
POWI: Postoperative wound infection
RCT: Randomized controlled trial
SPSS: Statistical Package for the Social Sciences
QALY: Quality adjusted life year
WIFI: Wound infections following implant removal
